# Promiscuous Diffusible Signal Factor Production and Responsiveness of the *Xylella fastidiosa* Rpf System

**DOI:** 10.1128/mBio.01054-16

**Published:** 2016-07-19

**Authors:** Michael Ionescu, Kenji Yokota, Elena Antonova, Angelica Garcia, Ellen Beaulieu, Terry Hayes, Anthony T. Iavarone, Steven E. Lindow

**Affiliations:** a Department of Plant and Microbial Biology, University of California, Berkeley, California, USA; b Department of Applied Biology and Chemistry, Tokyo University of Agriculture, Tokyo, Japan; c Biosciences Division, SRI International, Menlo Park, California, USA; d California Institute for Quantitative Biosciences (QB3), University of California, Berkeley, California, USA

## Abstract

Cell density-dependent regulation of gene expression in *Xylella fastidiosa* that is crucial to its switching between plant hosts and insect vectors is dependent on RpfF and its production of 2-enoic acids known as diffusible signal factor (DSF). We show that *X. fastidiosa* produces a particularly large variety of similar, relatively long-chain-length 2-enoic acids that are active in modulating gene expression. Both *X. fastidiosa* itself and a *Pantoea agglomerans* surrogate host harboring *X. fastidiosa* RpfF (*Xf*RpfF) is capable of producing a variety of both saturated and unsaturated free fatty acids. However, only 2-*cis* unsaturated acids were found to be biologically active in *X. fastidiosa*. *X. fastidiosa* produces, and is particularly responsive to, a novel DSF species, 2-*cis*-hexadecanoic acid that we term *Xf*DSF2. It is also responsive to other, even longer 2-enoic acids to which other taxa such as *Xanthomonas campestris* are unresponsive. The 2-enoic acids that are produced by *X. fastidiosa* are strongly affected by the cellular growth environment, with *Xf*DSF2 not detected in culture media in which 2-tetradecenoic acid (*Xf*DSF1) had previously been found. *X. fastidiosa* is responsive to much lower concentrations of *Xf*DSF2 than *Xf*DSF1. Apparently competitive interactions can occur between various saturated and unsaturated fatty acids that block the function of those agonistic 2-enoic fatty acids. By altering the particular 2-enoic acids produced and the relative balance of free enoic and saturated fatty acids, *X. fastidiosa* might modulate the extent of DSF-mediated quorum sensing.

## IMPORTANCE

*X. fastidiosa*, having a complicated lifestyle in which it moves and multiplies within plants but also must be vectored by insects, utilizes DSF-based quorum sensing to partition the expression of traits needed for these two processes within different cells in this population based on local cellular density. The finding that it can produce a variety of DSF species in a strongly environmentally context-dependent manner provides insight into how it coordinates the many genes under the control of DSF signaling to successfully associate with its two hosts. Since the new DSF variant *Xf*DSF2 described here is much more active than the previously recognized DSF species, it should contribute to plant disease control, given that the susceptibility of plants can be greatly reduced by artificially elevating the levels of DSF in plants, creating “pathogen confusion,” resulting in lower virulence.

## INTRODUCTION

The xylem-limited plant pathogen *Xylella fastidiosa* causes serious diseases of several important agricultural crops, including Pierce’s disease (PD) of grapevine and variegated chlorosis in citrus (CVC) (1, 2). *X. fastidiosa* is obligately transmitted from one plant to another by xylem sap-feeding insects. Like related *Xanthomonas* species, *X. fastidiosa* utilizes one or more signal molecules known as diffusible signaling factor (DSF) to regulate its behavior in a cell density-dependent manner (3, 4).

Previous studies implicated DSF-mediated cell-cell signaling in host switching by *X. fastidiosa*. Such signaling is apparently a cue that enables a subset of cells inside the plant to become preadapted to acquisition and transmission by insects to new host plants once a sufficiently high level of DSF is experienced. Acquisition of cells by insect vectors is strongly dependent on their ability to adhere to the walls of the insect’s foregut. DSF-mediated signaling regulates the transition from a nonadhesive, motile phenotype that allows systemic plant colonization to more adhesive cells that can form biofilms in insects and colonize insects (reviewed in reference 5). DSF induces the expression of many genes in *X. fastidiosa* (6, 7) including *hxfA* and *hxfB* that encode hemagglutinin-like proteins that are involved in cell-cell aggregation and biofilm formation (8). Since attachment-promoting traits in *X. fastidiosa* are incompatible with its movement inside the plant, DSF-producing grape plants were successfully employed to control PD by trapping the pathogen in a phenotype inconsistent with movement in plants, causing them to remain localized near the point of inoculation (9).

The DSF molecules that have been characterized are typically 2-*cis* enoic acids with a chain length of 12 to 14 carbons (10). To date, eight active DSF molecules have been reported in a variety of bacterial species. DSF (2-*cis*-11-methyldodecenoic acid), BDSF (2-*cis*-dodecenoic acid), CDSF [(2-*cis*,5-*cis*)-11-methyldodecadienoic acid], IDSF (2-*cis*-10-methyl-dodecenoic acid; also called DSF-II), 2-*cis*-9-methyldecenoic acid, and 2-*cis*-undecenoic acid were isolated from cultures of *Xanthomonas campestris* pv. campestris (11, 12, 13). DSF, BDSF, and CDSF were also isolated from *Xanthomonas oryzae* pv. oryzae (14).

BDSF was originally isolated from *Burkholderia cenocepacia* (15), while CDSF was originally reported to be produced by several *Burkholderia* species that were also reported to produce BDSF (16). DSF was found to be produced also by *Burkholderia multivorans* (16). 2-*cis*-Decenoic acid has been found in *Pseudomonas aeruginosa* (17). We previously isolated *X. fastidiosa* DSF (*Xf*DSF) (2-*cis*-tetradecenoic acid) from a grape strain of *X. fastidiosa* (18). A saturated acid molecule, (12-methyltetradecenoic acid) isolated from an *X. fastidiosa* CVC strain was proposed to be a DSF molecule (19), but it has not been shown to be biologically active.

DSFs are synthesized by RpfF, a unique crotonase that has both 3-hydroxyacyl-acyl carrier protein (ACP) dehydratase and thioesterase activity (20). RpfF first catalyzes the formation of a double bond between carbons 2 and 3 of a 3-hydroxyacyl moiety and then hydrolyzes the thioester bond with ACP to release a free acid. ^13^C-labeling experiments demonstrated that glucose acts as a substrate to provide a carbon element for DSF biosynthesis (21). Once DSF reaches a threshold concentration outside the cell, it activates its cognate receptor, RpfC, a hybrid membrane sensor kinase that phosphorylates the intracellular response regulator RpfG. RpfG then converts the intercellular signal into an intracellular signal through its cyclic di-GMP phosphodiesterase activity (22), which in turn, alters the expression of target genes (7, 23). In a previous study (24), we demonstrated that the DSF sensing mechanism in *X. fastidiosa*, unlike in *X. campestris*, is RpfF dependent; an *rpfF* deletion mutant could not sense externally applied DSF, while a strain harboring an *rpfF* variant (designated rpfF*) in which DSF synthesis was blocked via substitution of two glutamic acid residues with alanine residues (E141A and E161A), was able to sense and respond to DSF. This strain was the basis for an *X. fastidiosa*-based DSF sensor we designate the *Xf*DSF-biosensor strain.

The composition of the mixed DSF signals produced by *X. oryzae* was shown to be influenced by the composition of the culture media in which it grew (12, 14). In addition, while DSF or BDSF production has not been observed in *X. fastidiosa*, replacement of the native RpfF of *X. campestris* with that of *X. fastidiosa* (*Xf*RpfF) conferred production of DSF and BDSF as well as abundant *Xf*DSF (20). Therefore, we hypothesized that *Xf*RpfF, while exhibiting some degree of specificity in the production of the longer-chain *Xf*DSF, is relatively promiscuous compared to that of RpfF from *X. campestris*, and its products will be strongly influenced by the host in which it is being expressed and its environment. This suggests that a different compositional mixture of known DSF species and perhaps novel DSF species might be produced by *Xf*RpfF when grown under different conditions, such as in the xylem of host plants.

## RESULTS

### *Xf*RpfF produces a collection of free fatty acids.

**FIG 1:**
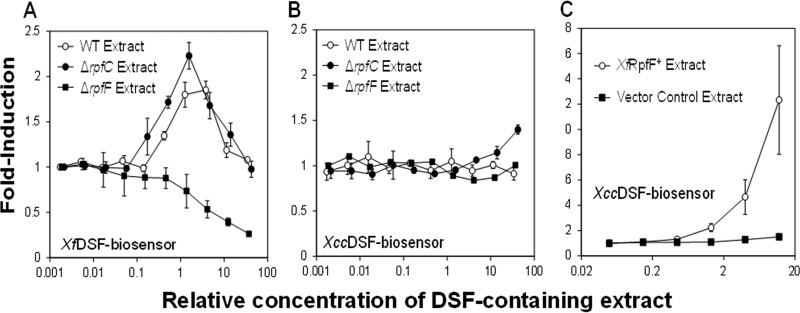
(A) Induction of the *Xylella fastidiosa*-based DSF biosensor strain (*Xf*DSF-biosensor strain) and (B) the *Xanthomonas campestris*-based DSF biosensor strain (*Xcc*DSF-biosensor strain) by various concentrations of DSF-containing culture extracts of *Xylella fastidiosa* strain. (C) Induction of the *Xcc*DSF-biosensor strain by extracts of *Pantoea agglomerans* 299R expressing *Xf*RpfF or harboring a vector only. The extract concentration reflects the volume of culture supernatant that would have been extracted with ethyl acetate to yield that added to 1 volume of assay medium.

In a previous study (18), we isolated and characterized a 14-carbon DSF species 2-*cis*-tetradecenoic acid (*Xf*DSF) that was produced and recognized by *X. fastidiosa*. In this study, *Xf*DSF was produced in the surrogate host *Pantoea agglomerans* 299R harboring *Xf*RpfF, and its isolation was guided by an *X. campestris*-based DSF-biosensor (4), which we designate the *Xcc*DSF-biosensor strain (Xcc stands for *X. campestris* pv. campestris) here. *Xf*DSF production in *X. fastidiosa* was then confirmed in an *rpfC* mutant strain that overproduces DSF (6, 18). Since the *Xcc*DSF-biosensor strain is more responsive to the DSF produced by *X. campestris* than to the *Xf*DSF produced by *X. fastidiosa* (18, 24), we hypothesized that other DSF molecules to which *X. campestris* would be unresponsive are produced by *X. fastidiosa*. In order to address this hypothesis, DSF production by a wild-type (WT) strain of *X. fastidiosa*, a Δ*rpfC* mutant which is an overproducer of DSF (6), and a Δ*rpfF* mutant blocked in DSF production was assessed using both the *Xcc*DSF-biosensor strain and the *Xf*DSF-biosensor strain. While the *Xf*DSF-biosensor strain was activated by DSF-containing extracts of cultures of the WT and the Δ*rpfC* mutant representing as little as 0.42 and 0.17 the concentration present in the original culture media, respectively (Fig. 1A), the *Xcc*DSF-biosensor strain was activated only by the DSF-containing extract of the Δ*rpfC* mutant that was 14-fold higher than that in the original culture media (Fig. 1B). No DSF could be detected by the *Xcc*DSF-biosensor strain in extracts of cultures of the WT strain even at high concentrations (Fig. 1B). Since the *Xf*DSF-biosensor strain responds to *Xf*DSF at 10-fold-lower concentrations than those perceived by the *Xcc*DSF-biosensor strain (18), the greater responsiveness of the *Xf*DSF-biosensor strain to extracts of cultures of *X. fastidiosa* than that of the *Xcc*DSF-biosensor strain suggested that DSF species that were not recognized by *X. campestris* were present in these extracts. While the *Xcc*DSF-biosensor strain was largely unresponsive to extracts of *X. fastidiosa* cultures, a strong response to extracts of cultures of *P. agglomerans* harboring *Xf*RpfF was seen (Fig. 1C), suggesting that the cellular environment of *Xf*RpfF determines the quantities and/or properties of the DSF species it produces. This crude extract was toxic to the *Xf*DSF-biosensor strain (not shown) and therefore could not be tested for active molecules in the *Xylella* system.

**FIG 2:**
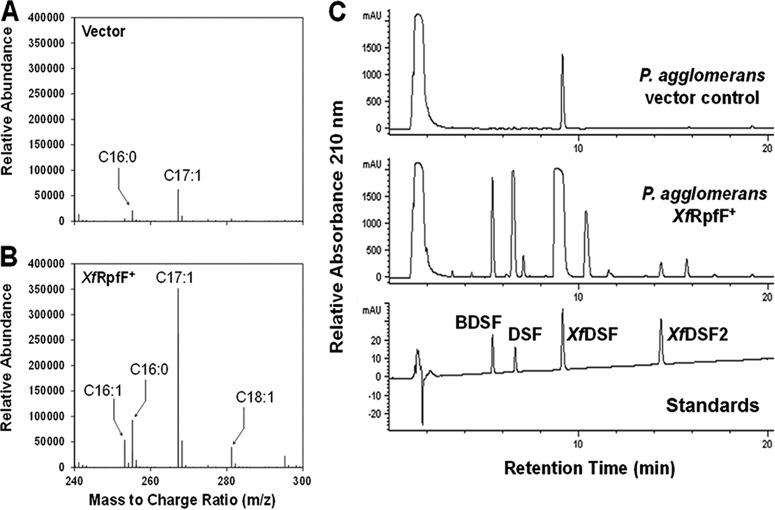
Electrospray ionization mass spectra (ESI-MS) of DSF-containing culture extracts of *Pantoea agglomerans* 299R harboring a vector only (A) or expressing *Xf*RpfF (B). (C) High-performance liquid chromatographs (HPLC) analysis of *Pantoea agglomerans* 299R harboring a vector only (top panel) or expressing *Xf*RpfF (middle panel) or solutions (250 µM) of synthetic standards.

DSF-containing extracts of WT *X. fastidiosa* and the Δ*rpfC* and Δ*rpfF* mutants, as well as *P. agglomerans* expressing *Xf*RpfF or harboring an empty vector, were analyzed by electrospray ionization mass spectrometry (ESI-MS) in a negative-ion mode to identify fatty acids that were expected to serve as signaling molecules. Free ionized fatty acids were identified by their mass-to-charge ratio (*m/z*). The accumulation of several saturated and unsaturated fatty acids was strongly dependent on *Xf*RpfF in both *X. fastidiosa* and *P. agglomerans* (Table 1; Fig. 2A and B). Five unsaturated fatty acids detected in *P. agglomerans* were apparently *Xf*RpfF-dependent, being 5-fold or more abundant in strains harboring *Xf*RpfF than in the vector control. These molecules could be tentatively identified as enoic acids from their *m/z* values: 225.185 (C_14:1_), 253.217 (C_16:1_), 267.232 (C_17:1_), 281.248 (C_18:1_), and 295.263 (C_19:1_) (Table 1). In addition, molecules with an *m/z* corresponding to DSF (*m/z* = 211.169) and BDSF (*m/z* = 197.154) were also found only in cells harboring *Xf*RpfF (Table 1), but their overall abundance and enrichment in the strain harboring *Xf*RpfF compared to the control strain was low. Three of these DSF-like molecules, C_16:1_, C_17:1_, and C_18:1_, were also found to be RpfF dependent in extracts of *X. fastidiosa* cultures, being in higher abundance in extracts of the Δ*rpfC* mutant than in the WT strain (Table 1) and greatly reduced in abundance in a Δ*rpfF* mutant. Interestingly, *Xf*DSF (C_14:1_) was not among the molecules produced by the WT *X. fastidiosa* strain, perhaps since the culture medium (PD3) used in this study was different than the periwinkle wilt GelRite medium (PWG) used previously (18). A low apparent abundance of C_14:1_ in extracts of the Δ*rpfC X. fastidiosa* strain (Table 1) probably accounts for the weak responsiveness of the *Xcc*DSF-biosensor strain to culture extracts of this strain, and suggested that one or more of the other putative enoic acids contributed to the responsiveness of the *Xf*DSF-biosensor strain (Fig. 1B).

**TABLE 1:** Abundance of putative fatty acid-derived molecular ions^a^

Mass/charge (*m/z*)^b^	Formula^b^	Predicted molecule	Abundance of putative fatty acid-derived molecular ions in^c^:				
			*P*. *agglomerans*		*X*. *fastidiosa* strains		
			*Xf*RpfF^+^	Vector	WT	Δ*rpfC*	Δ*rpfF*
197.154	C_12_H_21_O_2_	BDSF (C_12:1_)	1,460	635	0	0	0
211.169	C_13_H_23_O_2_	DSF (C_13:1_)	1,724	683	0	0	0
225.185	C_14_H_25_O_2_	*Xf*DSF (C_14:1_)	4,165	469	0	421	0
239.201	C_15_H_27_O_2_	C_15:1_	0	0	858	0	0
253.216	C_16_H_29_O_2_	*Xf*DSF2 (C_16:1_)	51,594	5,269	258,017	394,650	13,967
251.201	C_16_H_27_O_2_	C_16:2_	130	0	0	0	0
267.232	C_17_H_31_O_2_	C_17:1_	351,331	62,930	1,855	6,046	0
281.248	C_18_H_33_O_2_	C_18:1_	39,796	4,268	23,281	57,148	915
295.263	C_19_H_35_O_2_	C_19:1_	22,064	295	363	0	0
309.279	C_20_H_37_O_2_	C_20:1_	0	0	307	0	0
199.169	C_12_H_23_O_2_	C_12:0_	1,481	29	8,419	1,378	2,371
213.185	C_13_H_25_O_2_	C_13:0_	0	0	0	645	715
227.201	C_14_H_27_O_2_	C_14:0_	6,080	1935	0	0	0
241.216	C_15_H_29_O_2_	CVC-DSF (C_15:0_)	213	35	7,225	4,009	0
255.232	C_16_H_31_O_2_	C_16:0_	92,927	20,284	108,892	216,303	6,069
269.248	C_17_H_33_O_2_	C_17:0_	4,305	754	1994	16,567	0
283.263	C_18_H_35_O_2_	C_18:0_	1,896	608	22,145	71,704	830
297.279	C_19_H_37_O_2_	C_19:0_	0	0	0	0	0
311.295	C_20_H_39_O_2_	C_20:0_	0	0	543	8,187	0

a Abundance of putative fatty acid-derived molecular ions resolved by electrospray ionization-mass spectrometry (ESI-MS) analysis of DSF-containing culture extracts of *P. agglomerans* and *X. fastidiosa* strains.

b The m/z values and chemical formulas are of ionized molecules.

c Abundance of putative fatty acid-derived molecular ions (in relative arbitrary counts) resolved by ESI-MS analysis of DSF-containing culture extracts of *P. agglomerans* expressing vector only or expressing *Xf*RpfF (*Xf*RpfF^+^) and three *X. fastidiosa* strains, the wild-type *X. fastidiosa* strain and *rpfF* and *rpfC* deletion mutant strains.

It should be noted that fatty acids with identical *m/z* values could have different unsaturated sites or could be in a *trans* conformation rather than a *cis* conformation. For example, C_16:1_ and C_18:1_ could represent not only DSF-like molecules with *cis* unsaturated bonds at carbon atom 2 but also components of the bacterial membrane typically having unsaturated bonds in the middle of the aliphatic chain (reviewed in reference 25). However, given that the relative abundance of ions corresponding to C_16:1_ or C_18:1_ were 18- to 62-fold higher in extracts of the *X. fastidiosa* WT and Δ*rpfC* strains compared to that in the Δ*rpfF* strain, DSF-like 2-*cis*-enoic acid molecules apparently dominate these extracts with other, presumably membrane-derived molecules that are uncommon.

### *Xf*RpfF produces 2-*cis*-hexadecenoic acid.

In order to determine the structure and conformation of the *Xf*RpfF-dependent enoic acids discovered by ESI-MS, we employed reverse-phase high-performance liquid chromatography (HPLC) after separation on a C_18_ column, using selected synthetic 2-*cis* enoic acids as standards. Eight *Xf*RpfF-dependent molecules produced by *P. agglomerans* could be detected (Fig. 2C), while *X. fastidiosa* extracts did not contain enough material for such an analysis (not shown). Four compounds in extracts of *P. agglomerans* cultures coeluted with synthetic standards: BDSF (retention time [RT] = 5.5 min), DSF (RT = 7.1 min), *Xf*DSF (RT = 9.2 min), and 2-*cis*-hexadecenoic acid (RT = 14.3 min) (Fig. 2C). HPLC analysis enabled the relative concentrations of these molecules to be more readily determined than ESI-MS did. Of these four molecules, *Xf*DSF was present in the highest concentration (50 µM), while DSF, BDSF, and 2-*cis*-hexadecenoic acid were equally abundant. The other four molecules were isolated and tested for activity with the *Xf*DSF-biosensor strain (see Fig. S1 in the supplemental material). Only the compound with a retention time of 10.5 min activated the biosensor, and its analysis by ESI-MS revealed a molecule with an *m/z* of 251.20, corresponding to a 16-carbon enoic acid with two unsaturated sites. Nucleic magnetic resonance (NMR) analysis confirmed that one unsaturated site is at position 2 and in the *cis* conformation, but the position and conformation of the second unsaturated site could not be determined (data not shown).

Analysis of extracts of *X. fastidiosa* cultures and synthetic standard molecules using gas chromatography-mass spectrometry (GC-MS) was used to support the presence of 2-*cis*-hexadecenoic acid (C_16:1_) in the extract of the WT strain (see Fig. S2 in the supplemental material). Extracts of the WT and mutant strains were treated with boron trifluoride diethyl etherate (BF_3_ ⋅ OEt_2_) to esterify fatty acid derivatives, making them easier to detect by this method. While a peak with the same retention time as that of the synthetic C_16:1_ was observed in both the WT and Δ*rpfC* mutant extracts, the mass signature for C_16:1_ was below the detection limit at the concentrations found in the extracts. Other DSF-like molecules were not identified by GC-MS and were most likely present at concentrations too low to detect using this chromatography method.

Interestingly, small amounts of a compound that coeluted with *Xf*DSF (RT = 9.2 min) were detected in HPLC analysis of extracts of the *P. agglomerans* control strain harboring only the empty vector (Fig. 2C). DSF signaling has not been reported in this organism. A search for orthologous proteins to *Xf*RpfF in the draft genome of *P. agglomerans* 299R (26) revealed the presence of gene F385_3254, encoding a protein with 37% identity to that of *X. fastidiosa* RpfF (see Fig. S3 in the supplemental material). The *Xcc*DSF-biosensor strain exhibited a weak response to this extract when present in relatively high concentrations (at higher concentrations than those used in Fig. 1C), further suggesting that *P. agglomerans* 299R itself produces a small amount of a fatty acid species capable of DSF signaling activity.

### *Xf*RpfF-dependent unsaturated fatty acids are biologically active.

**FIG 3:**
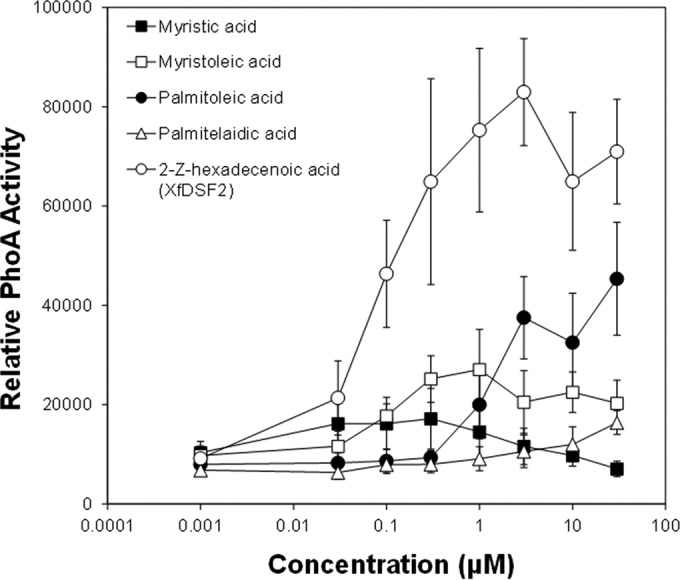
Alkaline phosphatase activity exhibited by the *Xylella fastidiosa*-based DSF biosensor strain (*Xf*DSF-biosensor strain) in cultures exposed to different concentrations of myristic acid (tetradecanoic acid), myristoleic acid (9-*cis*-tetradecenoic acid), palmitoleic acid (9-*cis*-hexadecenoic acid), palmitelaidic acid (9-*trans*-hexadecenoic acid), and 2-*cis*-hexadecenoic acid (*Xf*DSF) shown on the abscissa after 96-h incubation. The error bars represent the standard errors of the means.

**FIG 4:**
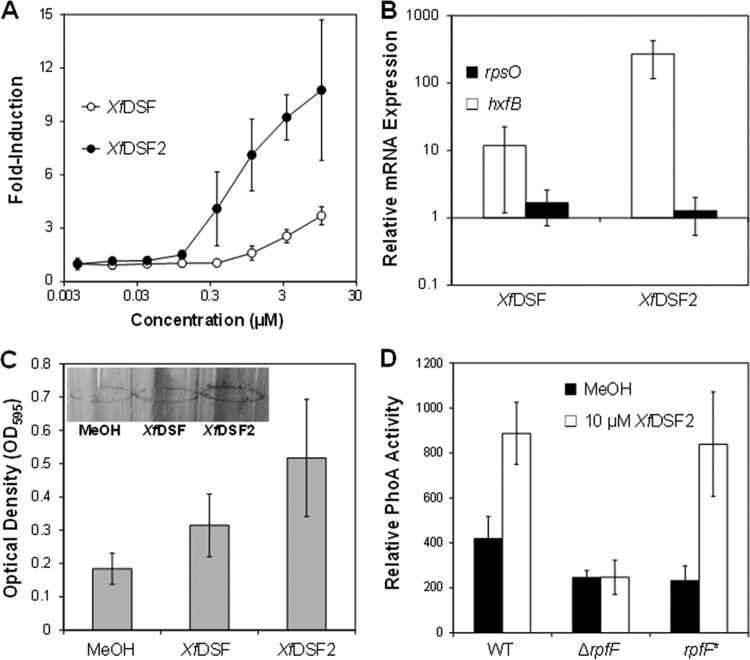
(A) Dose-dependent induction of the *Xylella fastidiosa*-based DSF biosensor (*Xf*DSF-biosensor strain) by various concentrations of *Xf*DSF and *Xf*DSF2. PhoA activity was measured at 72 h. (B) qRT-PCR analysis of *hxfB* expression as well as expression of the housekeeping gene *rpsO* in the *Xylella fastidiosa* rpfF* strain after 72 h or growth in PD3 broth supplemented with 10 µM *Xf*DSF or 10 µM *Xf*DSF2. *rpoD* and *rpsO* were used endogenous control genes. Values shown are the ratios of transcript abundance relative to that of cells exposed to a similar volume of MeOH alone. (C) Biofilm formation at the liquid-air interface of shaken glass tubes by the *Xylella fastidiosa* rpfF* mutant strain after 24 h of growth in PD3 in broth supplemented with 10 µM *Xf*DSF, 10 µM *Xf*DSF2, or MeOH alone as measured by a crystal violet assay. (D) Induction of the *hxfA′*::*phoA* transcriptional fusion in *Xylella fastidiosa* WT, Δ*rpfF*, and rpfF* strains by 10 µM *Xf*DSF2 as determined by alkaline phosphatase activity.

Since all demonstrably active DSF species detected thus far are 2-*cis*-unsaturated fatty acids (10, 11, 14, 15, 18), we posited that the *Xf*RpfF-dependent species C_14:1_, C_16:1_, C_17:1_, C_18:1_, and C_19:1_ would be 2-*cis*-tetradecenoic acid, 2-*cis*-hexadecenoic acid, 2-*cis*-heptadecenoic acid, 2-*cis*-octadecenoic acid, and 2-*cis*-nonadecanoic acid, respectively. Therefore, we tested the biological activity of a set of synthetic 2-*cis* enoic acids ranging in length from 2-*cis-*octenoic acid (C_8:1_) to 2-*cis*-eicosenoic acid (C_20:1_) using both the *Xf*DSF-biosensor strain and the *Xcc*DSF-biosensor strain. While the *Xcc*DSF-biosensor strain responded to 2-*cis-*enoic acids that varied in length from 10 to 14 carbons, the *Xf*DSF-biosensor strain responded to molecules with chain lengths of 12 to 18 carbons. 2-*cis*-Enoic acids with chain lengths of 8 to 11 carbons were toxic to *X. fastidiosa* but did not influence the growth of *X. campestris* (Table 2). While *X. fastidiosa* responded with the greatest sensitivity and intensity to 2-*cis*-hexadecenoic and 2-*cis*-heptadecenoic acids, *X. campestris* was much more responsive to DSF and 2-*cis*-tridecenoic acid. *X. fastidiosa* did not respond to any 14-carbon enoic acids with unsaturated sites other than at carbon atom 2 or those with a *trans* conformation rather than a *cis* conformation (Table 2; Fig. 3). It did respond, however, although only at higher concentrations, to 9-*cis*-hexadecenoic acid (Table 2; Fig. 3), but the activity of the *Xf*DSF-biosensor strain in response to a given concentration of this compound was only about half as great as to an equivalent concentration of 2-*cis*-hexadecenoic acid (Fig. 3). Since 2-*cis*-hexadecenoic acid was a natural product of *Xf*RpfF in *X. fastidiosa* (Table 1; see Fig. 2S in the supplemental material) and since *X. fastidiosa* is particularly responsive to it (Table 2; Fig. 4A), it is a novel *X. fastidiosa* DSF which we term *Xf*DSF2.

**TABLE 2:** Activity of various unsaturated fatty acids as signal molecules in *Xylella fastidiosa* and *Xanthomonas campestris*

Chain length	Orientation	Unsaturated site	Molecule name	*Xf*DSF-biosensor strain		*Xcc*DSF-biosensor strain	
				Response and/or minimum concn detected (µM)	Fold induction	Response or minimum concn detected (µM)	Fold induction
8	*cis*	2	2-*cis*-Octenoic acid	Toxic 30		No response	
9	*cis*	2	2-*cis*-Nonenoic acid	Toxic 1		No response	
10	*cis*	2	2-*cis*-Decenoic acid	Toxic 2		10	4.1
11	*cis*	2	2-*cis*-Undecenoic acid	Toxic 3		1	12.4
12	*cis*		2-*cis*-Dodecenoic acid (BDSF)	3	3.2	0.5	12.4
12	*trans*	2	2-*trans*-Dodecenoic acid	Toxic 6		3	7.9
13	*cis*	2	2-*cis*-Tridecenoic acid	Toxic		0.1	17.9
13	*cis*	2	2-*cis*-11-Methyldodecenoic acid (DSF)	3	17.5	0.05	17.9
14	*cis*	2	2-*cis*-Tetradecenoic acid (*Xf*DSF)	1	3.3	7	4.8
14	*cis*	5	5-*cis*-Tetradecenoic acid	No response		No response	
14	*cis*	6	6-*cis*-Tetradecenoic acid	No response		No response	
14	*cis*	9	9-*cis*-Tetradecenoic acid (myristoleic acid)	No response		No response	
14		0	Tetradecenoic acid (myristic acid)	No response			
15	*cis*	2	2-*cis*-Pentadecenoic acid	10	4.2	No response	
15		0	12-Methyltetradecanoic acid (CVC-DSF)	No response		No response	
16	*cis*	2	2-*cis*-Hexadecenoic acid (*Xf*DSF2)	0.15	8.9	No response	
16		0	Hexadecanoic acid	No response		No response	
16	*cis*	9	9-*cis*-Hexadecenoic acid (palmitoleic acid)	1	4.6	No response	
16	*trans*	9	9-*trans*-Hexadecenoic acid (palmitelaidic acid)	No response		No response	
17	*cis*	2	2-*cis*-Heptadecenoic acid	0.3	8.6	No response	
18	*cis*	2	2-*cis*-Octadecenoic acid	1	6.5	No response	
19	*cis*	2	2-*cis*-Nonadecenoic acid	No response		No response	
20	*cis*	2	2-*cis*-Eicodecenoic acid	No response		No response	

### *Xf*DSF2 is more active as a signaling molecule than *Xf*DSF.

*X. fastidiosa* responded to lower concentrations of *Xf*DSF2 than to *Xf*DSF (minimum detected concentrations of 0.15 µM versus 1.0 µM, respectively) as measured by the promoter activity of the *hxfA* gene in the *Xf*DSF-biosensor strain (Table 2; Fig. 4A). Furthermore, above these threshold levels, the alkaline phosphatase activity exhibited by the *Xf*DSF-biosensor strain at a given concentration was much higher in the presence of *Xf*DSF2 than in the presence of *Xf*DSF (Fig. 4A). The higher ability of *Xf*DSF2 to induce gene expression in *X. fastidiosa* was confirmed in measurements of the expression of the *hxfB* gene, encoding another hemagglutinin-like protein that is negatively controlled by *Xf*RpfF but located elsewhere in the genome. Expression of *hxfA* in the *X. fastidiosa* rpfF* mutant upon exposure to 10 µM *Xf*DSF2 was more than 10-fold higher than when exposed to 10 µM *Xf*DSF (Fig. 4B). Given that *Xf*DSF stimulates biofilm formation and increases the attachment of cells to the liquid-air interface of shaken cultures in glass tubes (15), we compared the apparent adhesiveness of cells of the rpfF* mutant in the presence of 10 µM *Xf*DSF or *Xf*DSF2 grown in this way. After as little as 24 h of incubation, the number of cells attached to the glass was 2.8- and 1.7-fold higher in the presence of *Xf*DSF2 and *Xf*DSF1, respectively, than in the control (Fig. 4C), indicating that *Xf*DSF2 more effectively induces the transition of planktonic to sessile cells. We previously also reported that expression of *hxfA* in the *X. fastidiosa* Δ*rpfF* deletion mutant was unresponsive to *Xf*DSF (20); this mutant also did not respond to *Xf*DSF2, while a high level of induction of *hxfA* was observed in the rpfF* mutant (Fig. 4D).

### Saturated fatty acids inhibit induction of the *Xf*DSF2-dependent *hxfA* promoter.

**FIG 5:**
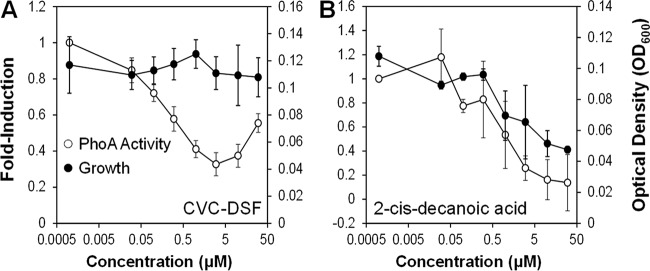
Suppression of the activity of the alkaline phosphatase activity exhibited by the *Xylella fastidiosa*-based DSF biosensor (*Xf*DSF-biosensor strain) by different concentrations of CVC-DSF (12-methyltetradecanoic acid) (A) and 2-*cis*-decanoic acid (B). PhoA activity was measured after 96 h of incubation and is shown as the proportion of that exhibited by control cultures to which no test material was added. Cell concentration (OD_600_) of cultures after incubation for 96 h (closed circles) is shown on the right ordinate.

**FIG 6:**
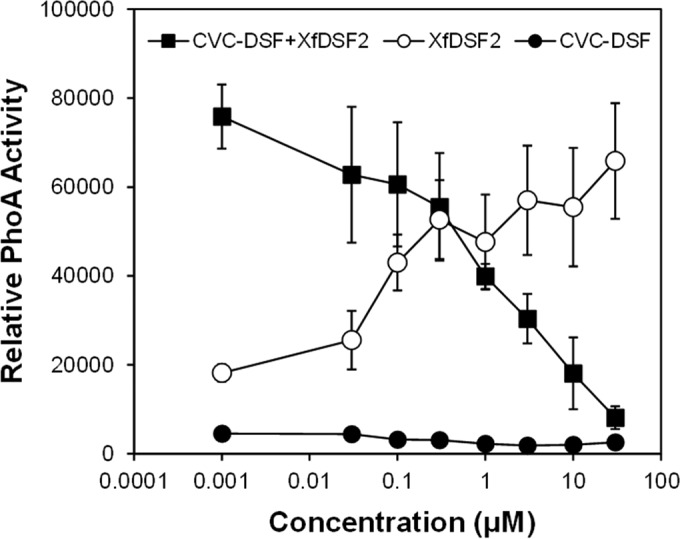
Alkaline phosphatase activity exhibited by the *Xylella fastidiosa*-based DSF biosensor (*Xf*DSF-biosensor strain) in cultures exposed to various concentrations of 2-*cis*-hexadecenoic acid (*Xf*DSF2), 12-methyltetradecanoic acid (CVC-DSF), or both 1 µM 2-*cis*-hexadecenoic acid and various concentrations of 12-methyltetradecanoic acid after 96-h incubation. The error bars represent the standard errors of the means.

**FIG 7:**
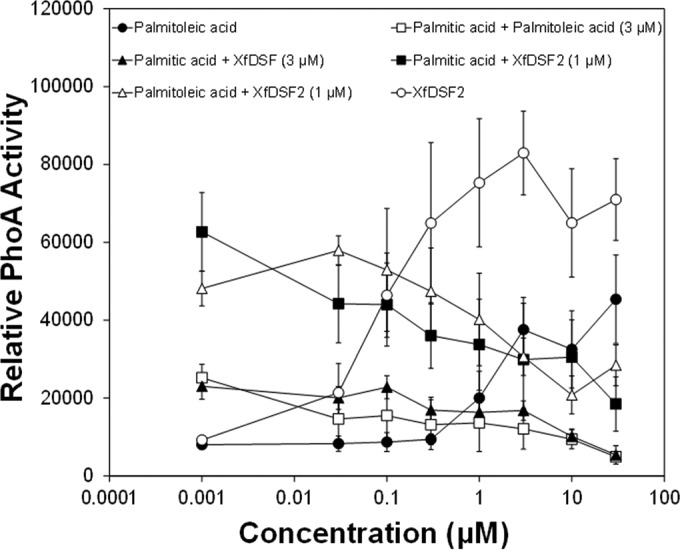
Alkaline phosphatase activity exhibited by the *Xf*DSF-biosensor strain in cultures exposed to various concentrations of fatty acids. The cultures were exposed to 9-*cis*-hexadecenoic acid (palmitoleic acid) or 2-*cis*-hexadecenoic acid (*Xf*DSF2) or to both 3 µM 9-*cis*-hexadecenoic acid and various concentrations of hexadecanoic acid (palmitic acid), 3 µM 2-*cis*-tetradecenoic acid (*Xf*DSF) and various concentrations of hexadecanoic acid), 1 µM 2-*cis*-hexadecenoic acid (*Xf*DSF2) and various concentrations of hexadecanoic acid, 1 µM 2-*cis*-hexadecenoic acid and various concentrations of 9-*cis*-hexadecanoic acid shown on the abscissa after 96-h incubation. The error bars represent the standard errors of the means.

While saturated fatty acids conferred no induction of *hxfA* in the *Xf*DSF-biosensor strain (Table 2), some fatty acids, such as tetradecanoic acid (myristic acid) and CVC-DSF, suppressed the basal activity of the *Xf*DSF-biosensor strain in a dose-dependent manner without affecting its growth (Fig. 5A). This phenomenon was distinct from that seen with short-chain 2-*cis*-enoic acids, such as 2-*cis*-decenoic acid that both inhibited *hxfA* activity and suppressed growth of the *Xf*DSF-biosensor strain in a dose-dependent manner (Fig. 5B). The latter molecules were therefore termed “inhibitory fatty acids” to distinguish them from the “antagonistic” saturated fatty acids that merely reduced the expression of DSF-dependent genes in *X. fastidiosa*. Not only did CVC-DSF reduce the basal expression of the *hxfA* promoter in the *Xf*DSF-biosensor strain (Table 2), it also reduced the responsiveness of *X. fastidiosa* to DSF species such as *Xf*DSF2. The activity of the *Xf*DSF-biosensor strain in the presence of 1 µM *Xf*DSF2 decreased proportionally as the concentration of CVC-DSF added to cultures increased (Fig. 6). At relatively high concentrations of CVC-DSF (10 µM), no apparent induction of the *hxfA* promoter was observed, even in the presence of sufficient *Xf*DSF2 to elicit strong induction of *hxfA*. At equal molar concentrations of *Xf*DSF2 and CVC-DSF, the apparent expression of the *hxfA* promoter, as measured by the alkaline phosphatase activity exhibited by the *Xf*DSF-biosensor strain, was reduced about 50% (Fig. 6). Induction of the *hxfA* promoter by various agonistic fatty acids, including *Xf*DSF, *Xf*DSF2, and 9-*cis*-hexadecenoic acid, could all be suppressed in the presence of saturated fatty acids such as hexadecanoic acid (palmitic acid). In all cases, the addition of palmitic acid to cultures containing one of these various DSF species decreased the alkaline phosphatase activity exhibited by the *Xf*DSF-biosensor strain in a dose-dependent manner (Fig. 7). As was seen with mixtures of CVC-DSF and *Xf*DSF2, equal molar concentrations of palmitic acid added to culture media with *Xf*DSF2 reduced the apparent expression of the *hxfA* promoter about 50%. Likewise, induction of *hxfA* by *Xf*DSF was reduced about 50% when an equal molar concentration of palmitic acid was added to cultures (Fig. 7). Curiously, even though 9-*cis*-hexadecenoic acid (palmitoleic acid) was able to induce *hxfA* expression at relatively high concentrations, it also reduced the induction of *hxfA* in the presence of *Xf*DSF2 by ca. 3-fold when added at equal molar concentrations to culture media (Fig. 7). Thus, the regulation of genes dependent on *Xf*RpfF and thus, DSF-mediated signaling in *X. fastidiosa*, appears to be conferred by the presence of a variety of similar, relatively long-chain unsaturated fatty acids. Furthermore, apparently competitive interactions can occur between various saturated and unsaturated fatty acids that are not effective inducers of DSF-dependent gene expression to block the function of those unsaturated fatty acids that can successfully interact with DSF receptors to modulate gene expression.

## DISCUSSION

Structurally confirmed members of the DSF family are all fatty acids that are unsaturated at carbon atom 2, a feature shown to be important for signaling in *Xanthomonas* and *Burkholderia* species (10, 20). *X. fastidiosa* RpfF, like the DSF synthases of other bacterial species, is a unique member of the corotonase family that produce 2-*cis* enoic acids. In this work we present evidence that *X. fastidiosa* RpfF produces and responds to exceptionally long-chain DSF species such as 2-*cis*-hexdecenoic acid, that we term *Xf*DSF2. Some evidence from mass spectrometry suggests that *X. fastidiosa* RpfF might produce even longer 2-*cis* enoic acids of 17 and 18 carbons. However, while *X. fastidiosa* is quite responsive to these two enoic acids (Table 2), insufficient amounts were recovered from culture media to confirm their levels.

While short- and medium-chain-length DSF species, such as BDSF and *Xf*DSF, can be predicted from their partition coefficient (log K_ow_ = 4.78 and 5.77, respectively, where K_ow_ is the octanol-water partition coefficient; http://www.chemspider.com) to be sufficiently soluble in water (23 µM and 4.2 µM, respectively) to account for their ability to act as signaling molecules at concentrations near 1 µM, *Xf*DSF2 is more hydrophobic and is estimated to have a solubility of only about 0.5 µM (log K_ow_ = 6.58). However, the *X. fastidiosa* DSF-biosensor strain exhibited a progressive dose-dependent response when *Xf*DSF2 was added to culture media at concentrations greater than 0.5 µM (Fig. 3 and 4A), indicating that it was bioavailable. These observations raise the question as to how such an apparently insoluble signal molecule such as *Xf*DSF2 can be acquired by cells and subsequently interact with the DSF sensor RpfC. We hypothesize that since DSF species, particularly *Xf*DSF2, are hydrophobic, they would interact with hydrophobic matrices such as bacterial membranes. Exogenously supplied DSF might thus be inserted into membranes where they are then distributed from one cell to another by contact or by sharing outer membrane vesicles shed by cells. While *X. fastidiosa* is a prolific producer of outer membrane vesicles (27), further work will be needed to address whether such vesicles play a role in DSF-mediated cell-cell signaling, as has been suggested for PQS (*Pseudomonas* quinolone signal) in *Pseudomonas aeruginosa* (28). Since *X. fastidiosa* lives exclusively within either xylem vessels or the mouthparts of insect vectors, both sites of rapid fluid flow, an extracellular signal molecule would be expected to be removed from this habitat if it were freely soluble. The relatively insoluble enoic acids such as *Xf*DSF and *Xf*DSF2 to which *X. fastidiosa* is most responsive are therefore less likely to be lost from the producing cells.

*Xf*RpfF is a promiscuous enzyme that has the potential to produce a variety of DSF molecules. As has been suggested from other studies of *X. campestris* (14), the products of *X. fastidiosa* RpfF are very dependent on the host environment in which it is expressed. For instance, *Xf*DSF was isolated from *X. fastidiosa* grown on PWG in a previous study (18), but when *X. fastidiosa* was grown on PD3 plates in this study, we could not detect *Xf*DSF in the extracts, indicating that the environment in which *Xf*RpfF functions determines the DSF species that it produces. This suggests that the dominant DSF species produced by bacteria in their natural habitat might be different than those produced in a given *in vitro* environment. Presumably such patterns of DSF production maximizes fitness by enabling appropriate responses to a given habitat.

It was reported (29) that in *Stenotrophomonas maltophilia*, RpfF plays a role in the biosynthesis of eight fatty acids, two of which are saturated fatty acids while six are unsaturated fatty acids (all six with double bonds at position 2). Similarly, we report here that the accumulation of several saturated and unsaturated fatty acids is dependent upon RpfF. Since RpfF is a bifunctional crotonase having both dehydratase and thioesterase activities (20), this observation can be explained if some of its substrates are cleaved without being first dehydrated. In *Burkholderia cenocepacia*, the RpfF homolog has been reported to catalyze the *in vitro* cleavage of acyl-ACP thioester bonds to yield a holo-ACP and a free fatty acid, a process independent of its dehydratase activity (20). Since the thioester bond is required for the formation of the unsaturated bond at carbon atom 2 (30), thioester cleavage would abort the dehydratase reaction and result in the release and accumulation of free saturated fatty acids. As such molecules are produced *in vivo* in *X. fastidiosa*, we hypothesize that they play a role in modulating cell-cell signaling. We report here that CVC-DSF and other saturated fatty acids that do not activate *hxfA* or other DSF-responsive genes in *X. fastidiosa* antagonize DSF-mediated signaling in *X. fastidiosa*. This antagonism is apparently not associated with any toxicity to the cells and thus growth inhibition. Instead, such saturated molecules appear to compete directly with 2-enoic acids for DSF receptors such as RpfC, since the responses to various enoic acids were reduced in a dose-dependent manner by an equal concentration of such molecules. This signaling antagonism did not appear to be very specific, as the response to all enoic acids investigated could be blocked by a given saturated fatty acid (Fig. 6 and 7), and a given saturated fatty acid, such as palmitic acid, could block the response to more than one enoic acid, such as *Xf*DSF, *Xf*DSF2, and palmitoleic acid (Fig. 7).

It is tempting to speculate that the role of RpfB, which has been shown to act as a fatty acyl-coenzyme A (CoA) ligase that counteracts RpfF thioesterase activity by catalyzing the uptake and activation of free fatty acids to yield acyl-CoAs (31), is to alter the relative abundance of fatty acids in the cell. Not only would RpfB restore fitness to cells by restoring some ability to synthesize membrane lipids in cells in which RpfF was active, but it could also play a role in modulating the composition of free fatty acids present in the cell if they were a selective substrate for RpfF. It was recently reported that in *X. campestris*, RpfB increased DSF and BDSF turnover (32). That is, by altering the relative balance of free enoic and saturated fatty acids in *X. fastidiosa* DSF-mediated signaling, RpfB could modulate the extent of quorum sensing. Given that *X. fastidiosa* both produces and is responsive to a particularly large range of enoic acids, the suggestion that it may have more than one receptor for such signal molecules (6) offers the possibility of having a contextual response to increasing cell density. For example, it is apparent from this study that different enoic acids are produced under different culture conditions, and thus, they probably also differ under the various conditions experienced in plant and insect hosts. Furthermore, the output of quorum sensing might also be modulated by the activity of RpfB (33). It is intriguing that unlike other bacteria capable of DSF-based cell signaling such as *X. campestris*, *rpfB* is located elsewhere from *rpfF* in the genome, suggesting that its expression might be independent of that of *rpfF*, unlike in other bacteria in which they are found together in an operon. The fact that RpfB mutants of *X. fastidiosa* are more deficient in traits enabling insect colonization and transmission to new host plants than virulence to plants (34) supports the conjecture that RpfB modulates the abundance of DSF species to modulate gene expression.

The complex lifestyle of *X. fastidiosa* in which the traits that are required for it to move and multiply within the xylem vessels of plants are incompatible with those required for its acquisition from plants by insect vectors may also have led to its apparently more versatile DSF-mediated cell signaling system. As a xylem-limited colonist of plants, *X. fastidiosa* would encounter relatively few other bacteria in this habitat, since the endophytic population size of most plants is quite low (35). As such, *X. fastidiosa* may uncommonly encounter other bacteria that produce fatty acid signal molecules. In contrast, species such as *X. campestris* which has a prominent epiphytic stage on plants might commonly encounter DSF-producing bacteria. Because of this putative chemical isolation, *X. fastidiosa* may not have been under pressure to restrict the interactions of its DSF receptors only to the various DSF species that it produces. Furthermore, as noted above, it might need to be able to flexibly interact with a suite of signal molecules to enable appropriate varied responses to different environmental conditions. While there has not been extensive study of the diversity of bacteria that produce DSF-like signal molecules, an increasing list of bacterial species capable of producing DSF (36, 37, 38) and the finding here that the common plant epiphyte *P. agglomerans* can apparently produce at least some *Xf*DSF (Fig. 2C) and harbors an apparently functional homolog to *X. fastidiosa* RpfF suggest that DSF-mediated signaling may be more common than previously thought. Various enoic acids thus have the potential to participate widely in interspecies interactions, a topic worthy of further investigation.

## MATERIALS AND METHODS

### Bacterial strains and plasmids.

The bacterial strains and plasmids used in this study are listed in Table 3. The *Xf*DSF-biosensor strain (previously designated *X. fastidiosa* rpfF*-XfHA biosensor [24]) consists of the rpfF* mutant (E141A E161A) that exhibits blocked DSF synthesis but can still sense externally applied DSF) harboring p*Xf*HA (*hxfA*′::*phoA*). The *X. campestris*-based DSF-biosensor strain (*Xcc*DSF-biosensor) (4) consists of strain 8523 (*rpfF* mutant) harboring pKLN55 (*engXCA′*::*gfp*). *P. agglomerans* harboring plasmid pVSP61-*rpfF* (18) and *X. campestris* and *P. agglomerans* cells were grown on King’s B medium (KB) (39). Inoculum of *X. fastidiosa* was grown on periwinkle wilt GelRite medium (PWG medium) plates for 5 to 7 days before transfer to PD3 broth (40). Antibiotics were added to a final concentration of 50 µg ml^−1^ for kanamycin and 15 µg ml^−1^ for gentamicin. All cultures were grown on 28°C in the dark.

**TABLE 3:** Bacterial strains and plasmids used in this study

Bacterial strain or plasmid	Relevant genotype or characteristic(s)	Reference
Bacterial strains		
*X. fastidiosa*Temecula1	Wild type; ATCC 700964	
Dif7	*X. fastidiosa* Temecula1Δ*rpfF* (markerless)	6
Rpf*	*X. fastidiosa* Temecula1 rpfF* (E141A E161A) (Kan^r^)	24
MIX3	*X. fastidiosa* Temecula1 Δ*rpfC* (Kan^r^)	This study
8523	*X. campestris* pv. *campestris* 8004 *rpfF*::Tn*5lac* (Kan^r^)	3
*Pantoeaagglomerans*299R	Wild type	41
*Xcc*DSF-biosensor	8523 bearing pKLN55	4
*Xf*DSF-biosensor	MIX2 bearing p*Xf*HA	18
Plasmids		
pFXF*kan*	pUC19 Kan^r^ [*aph*(3′)II]	24
pVSP61	pVS1 and pACY184 Ori Kan^r^	42
pFXF7	pFXF*kan* “rpfG-kan^R^-rpfF”	This study
pVSP61-*rpfF*	pVSP61 *kan′*::*rpfF* (*rpfF* of *X. fastidiosa*)	18
pKLN55	pVSP61 *Xanthomonas campestris* pv. campestris *engXCA′*::*gfp*	4
p*Xf*HA	pBBR1MCS-5 *hxfA′*::*phoA*	18

### Extraction of DSF from bacterial cultures.

DSF was extracted from *P. agglomerans* strains grown for 48 h in 1 to 3 liters of KB broth that was shaken at 200 rpm at 28°C. The pH of the medium was then adjusted to 4.0 (14), and an equal volume of water-saturated ethyl acetate (EtOAc) was added and mixed for 10 min. The EtOAc fraction was then separated from the medium using a separatory flask and concentrated by evaporation using a Rotavapor R evaporator (Büchi, Switzerland). The dried residues were dissolved in 1 to 3 ml methanol (MeOH).

DSF was extracted from *X. fastidiosa* strains grown for 2 weeks at 28°C on PD3 agar (150 plates; 3 liters). The medium containing the cells was sliced into 8-mm^3^ cubes and mixed for 2 h in an equal volume of EtOAc. The EtOAc was decanted by filtration through cheesecloth, and the decanted contents were concentrated *in vacuo* as described above. The dried residues were dissolved in 3 ml MeOH.

### Measurement of DSF biological activity in *X. campestris* pv. campestris.

DSF, BDSF, CVC-DSF, and myristic acid were purchased from Sigma Aldrich, dissolved in MeOH to a concentration of 100 mM and stored at −20°C. DSF-containing culture extracts or synthetic molecules in MeOH were added in MeOH to wells of Falcon 24-well tissue culture plates (Becton Dickinson, USA), and the MeOH was allowed to escape. An equal volume of MeOH was added to some wells as a negative control. Warm (60°C) KB agar containing kanamycin (2 ml) was then added to each well. Inoculum of the *Xcc*DSF-biosensor strain was grown for 2 days on KB containing kanamycin and suspended in 10 mM phosphate buffer (pH 7.4), and the optical density at 600 nm (OD_600_) was adjusted to 0.1 using a Spectronic 21D spectrophotometer (Milton Roy, USA), and 5-µl drops were spotted onto each well. After 2 days of incubation at 28°C, cells were collected and suspended in 0.2 ml of 10 mM phosphate buffer to a final cell density of 0.2 to 0.3 measured at OD_600_ in Falcon 96-well tissue culture plates (Becton Dickinson). For each well, both relative fluorescence units (RFU) (excitation wavelength, 485 nm; emission wavelength, 515 nm) and cell density measured as OD_600_ were recorded using a Synergy 2 plate reader (BioTek, USA), and green fluorescent protein (GFP) fluorescence was normalized as RFU OD_600_^−1^.

### Measurement of DSF biological activity in *X. fastidiosa.*

Inoculum of the *Xf*DSF-biosensor strain was grown for 5 or 6 days at 28°C on PWG plates containing gentamicin prior to suspension in PD3 broth containing gentamicin (final OD_600_ of 0.05) and various amounts of synthetic DSF molecules or DSF-containing culture extracts. MeOH only was used as a control, and its concentration in the medium was always 0.01%. Samples were then distributed (800 µl per well) into 48-well clear tissue culture plates (Becton Dickinson) with six replicates. After 96-h incubation at 28°C without shaking, the alkaline phosphatase activity (PhoA activity) was quantified (43). The plate was centrifuged for 10 min at 2,254 × *g* in an Eppendorf model 5804 centrifuge (Eppendorf, Germany), the growth medium was removed by aspiration, and the cells were resuspended in 0.4 ml of 10 mM Tris base (pH 8.0) containing 10 mM MgSO_4_. The cells were pelleted again by centrifuging for 10 min at 2,254 × *g*, resuspended in 0.4 ml of 1 M Tris base (pH 8.0) containing 0.4 mM ZnCl_2_, and the OD_600_ in each well was measured. The cells were then disrupted by adding 10 µl of 0.1% sodium dodecyl sulfate (SDS) and 10 µl chloroform to each well followed by 5 min of shaking (200 rpm) at room temperature. Sixty microliters of 1 M Tris base (pH 8.0) containing 0.4 mM ZnCl_2_ supplemented with 100 µM fluorescein diphosphate (AnaSpec, USA) stock solution was then added to each well, and fluorescence (excitation wavelength, 485 nm; emission wavelength, 515 nm) was measured at 2-min intervals for 30 min. OD_600_ and fluorescence were both measured using a Synergy 2 plate reader (Biotek, USA). Enzyme activity was calculated as the rate of increase of fluorescence over time divided by the cell density.

### Electrospray ionization mass spectrometry (ESI-MS).

DSF-containing culture extracts were analyzed using an LTQ Orbitrap XL mass spectrometer equipped with an electrospray ionization (ESI) source (Thermo Fisher Scientific, Waltham, MA, USA). Mass spectra were recorded in the negative-ion mode over the *m*/z range from 100 to 500 using the Orbitrap mass analyzer, in profile format, with a resolution setting of 100,000 (as measured at *m*/*z* = 400). Mass spectra were processed using Xcalibur software (version 2.0.7 SP1; Thermo Fisher Scientific).

### Synthesis of 2-*cis*-hexadecenoate.

Still-Gennari reagent (2.00 ml, 8.47 mmol, 1.2 equivalents [equiv]) was added to a solution of 1,4,7,10,13,16-hexaoxacyclooctadecane (18-crown-6) (7.45 g, 4 equiv) in tetrahydrofuran (THF) (60 ml) at room temperature. The mixture was cooled to −78°C, and a solution of potassium hexamethyldisilazide in toluene (17.0 ml of 0.5 M solution, 1.2 equiv) was added dropwise over 5 min. The mixture turned bright orange and was stirred for 45 min. A solution of tetradecanal (1.50 g, 7.06 mmol, 1.0 equiv) in THF (10 ml) was added dropwise over 10 min. The reaction mixture was stirred at −78°C for 3 h. The reaction was quenched by the addition of a saturated solution of NH_4_Cl (50 ml). The layers were separated, and the aqueous layer was extracted with ethyl acetate (extracted three times with 50 ml). The combined organics were washed with brine (once with 50 ml), the resulting liquid was dried over Na_2_SO_4_, and the volatiles were removed *in vacuo*. The crude oil was purified using flash column chromatography using a 5% to 10% gradient of ethyl acetate in hexane. The ethyl ester was isolated as a colorless oil (835 mg, 41%). The ester (806 mg, 2.85 mmol, 1.0 equiv) was dissolved in THF (10 ml). Solid LiOH (411 mg, 17.1 mmol, 6.0 equiv) was dissolved in H_2_O (10 ml) and added to the ester solution. The biphasic mixture was stirred vigorously at 60°C for 12 h at which point the reaction was complete by thin layer chromatography. The mixture was acidified with a 10% solution of HCl to a pH of 2. The mixture was extracted with ethyl acetate (three times with 30 ml). The combined organics were washed with brine (once with 20 ml), the resulting liquid was dried over Na_2_SO_4_, and 2-*cis*-hexadecenoic acid was isolated as a white solid (678 mg, 93%).

### Reverse-phase HPLC analysis.

DSFs were detected and quantified by reverse-phase high-performance liquid chromatography (HPLC) using an Agilent Technologies 1200 series high-performance liquid 150 chromatography system (Agilent Technologies, USA) as follows. Five microliters of the sample was injected into an HPLC column (Ascentis Express C_18_ column [150 mm long with an inner diameter of 4.6 mm]; Supelco, USA), which was eluted at a flow rate of 1 ml min^−1^ with two solvent gradients containing 0.1% trifluoroacetic acid, water (eluent A) or methanol (eluent B), at 50°C. The gradient conditions were as follows: starting at 20% eluent A and 80% eluent B, eluent A was linearly decreased to 10% by increasing the amount of eluent B over 20 min, and the eluent was monitored at 210 nm. DSF concentration in crude extracts was determined based on their peak area and calculated from standard curves generated for each of the DSF species using synthetic forms.

### GC-MS.

Gas chromatography-mass spectrometry (GC-MS) analysis of acids present in natural extracts was performed by derivatizing the acids into methyl esters. To confirm the presence of *Xf*DSF2 in natural extracts, a synthetic standard was used. DSFs were detected by GC-MS using an Agilent (HP) model 6890N GC with a 5973 mass selective detector. A vial containing 25.0 µl of each natural extract dissolved in MeOH (as prepared in “Extraction of DSF from bacterial cultures” above) was diluted with 25.0 µl of MeOH. To each vial was added 100.0 µl of a 10% (wt/wt) solution of BF_3_ ⋅ MeOH (commercially available from Sigma Aldrich). Esterification was carried out for 30 min at 45°C. After the reaction, 150.0 µl and 300.0 µl of hexanes were added to each vial, and the vial was vortexed for 20 s. The organic layer was extracted and dried under a stream of argon gas. Isolated solids were resuspended in 50.0 µl in methyl cyanide (MeCN) (acetonitrile) and analyzed by GC-MS.

To confirm the presence of *Xf*DSF2 in natural extracts, a 5.00 mM stock solution of synthetic 2-*cis*-hexadecenoic acid in MeOH was prepared. To a vial containing 25.0 µl of each natural extract dissolved in MeOH (as prepared in “Extraction of DSF from bacterial cultures” above) was added 25.0 µl of the 5.00 mM stock solution of synthetic 2-*cis*-hexadecenoic acid, followed by addition of 100.0 µl of a 10% (wt/wt) solution of BF_3_ ⋅ MeOH. Esterification was carried out for 30 min at 45°C. After the reaction, 150.0 µl and 300.0 µl of hexanes were added to each vial, and the vial was vortexed for 20 s. The organic layer was extracted and dried under a stream of argon gas. Isolated solids were resuspended in 50.0 µl in MeCN and analyzed by GC-MS.

Both the natural and spiked extracts were compared to a blank sample which was prepared by the use of 50.0 µl of pure MeOH treated as described above. The molecular ion for the methyl ester at 268 *m*/*z* was not detected by this ionization method, so the fragments at 237 and 171 *m*/*z* were used to identify *Xf*DSF2 in natural extracts.

### Quantification of gene expression with real-time PCR.

Gene expression analysis was performed on rpfF* mutant *X. fastidiosa* strain MIX2 (Table 3) subjected to experimental materials in a procedure similar to that employed for measurement of PhoA activity. Cells were harvested from PWG plates after 7 days of growth and suspended in 120-ml PD3 broth to an OD_600_ of 0.05. The medium was then divided into 3 equal parts of 40 ml that was then supplemented with 10 µM *Xf*DSF1, 10 µM *Xf*DSF2, or MeOH alone as a control. The samples were then distributed into three 48-well clear tissue culture plates (800 µl per well) and incubated at 28°C without shaking for 3 days. Total RNA was isolated using TRIzol extraction as follows. Cells were collected into a 50-ml Falcon tube and centrifuged for 10 min at 2,254 × *g* in an Eppendorf model 5804. The pellet was resuspended in 1-ml RNAlater RNA stabilization solution (Ambion, TX, USA) and transferred to a 2-ml Eppendorf tube. The cells were harvested again and resuspended in 1-ml TRIzol reagent (Ambion), mixed vigorously, and incubated until completely lysed at 60°C (ca. 5 min). Chloroform (200 µl) was added and mixed well, and the tubes were allowed to stand on the bench for 5 min before being subjected to 10 min of centrifugation at 20,800 × *g*. The clear upper phase was then collected (ca. 450 µl) and mixed with 500 µl isopropanol. The tubes were incubated for 30 min at −80°C and then centrifuged for 30 min at 20,800 × *g* at 4°C. The white pellet containing nucleic acids was washed twice with 70% cold ethanol (EtOH), and the pellet was air dried and suspended in 50 µl nucleic acid-free double-distilled water.

DNA was eliminated using Turbo DNase (Ambion, TX, USA). RNA samples were stored at −80°C, and fresh 1-µg aliquots were used for cDNA synthesis before each analysis, using 3 µg of random hexamers and Superscript II reverse transcriptase (Life Technologies, USA) according to the manufacturer’s instructions. Quantitative PCR was performed in an ABI PRISM 7100 sequence detector system (Applied Biosystems, USA). Detection of PCR products was done by measuring the increase in fluorescence produced upon binding of SYBR green dye (Qiagen, Germany) to double-stranded DNA. Both *rpoD* and *rpsO* were used as endogenous control genes to normalize gene expression. The primers for the *hxfB*, *rpoD*, and *rpsO* genes have been previously described (24). To ensure that the threshold cycle (*C_T_*) values obtained were from a single PCR product, melting curve analysis was run after each analysis. Relative expression (RQ) was calculated from the threshold cycle (*C_T_*) as follows: d*C_T_* = *C_T_* (target gene) − *C_T_* (endogenous control) where d*C_T_* is the change in the *C_T_* and *C_T_* (target gene) is the threshold cycle of the target gene; dd*C_T_* = d*C_T_* (treatment) − d*C_T_* (reference); RQ = 2^(−ddCT)^. RQ values (ratios between two compared samples) are presented as means ± standard deviations from three biological replicates of quantitative reverse transcription-PCR (qRT-PCR) assays performed in triplicate.

### Biofilm assay.

The effects of *Xf*DSF and *Xf*DSF2 on the biofilm formation capacity of the *X. fastidiosa* rpfF* mutant were determined in cells growing in PD3 broth cultures. Cells were grown on PWG plates for 7 days, resuspended in PD3 broth to an OD_600_ of 0.05, and added (2 ml) to PD3 broth cultures containing a final concentration of 10 µM of either 2-*cis-*tetradecenoic acid or 2-*cis*-hexdecenoic acid or an equal volume of MeOH alone as a control. The glass tubes, containing 2 ml of a culture, were shaken (200 rpm) for 24 h in glass tubes at 28°C during which time a visible biofilm formed at the liquid-air interface. The medium, containing unattached cells, was then removed by aspiration, the tubes were washed three times with tap water to remove unattached cells, and the biomass attached to the glass wall was stained with 2 ml of 1% crystal violet (CV) for 10 min. Excess CV was removed by washing the tubes three times with tap water, and the retained CV was dissolved in 1 ml of 95% ethanol and quantified by measuring absorbance at 595 nm (Spectronic 21D spectrophotometer; Milton Roy, USA).

## SUPPLEMENTAL MATERIAL

Figure S1  Induction of the *Xf*DSF-biosensor strain by either 2 or 10 µl of a purified fraction of a DSF-containing extract of *P. agglomerans* 299R expressing *X. fastidiosa* RpfF (fractions named based on their retention times in minutes in HPLC [HPLC RT]). White bars represent induction of the sensor by 10 µM *Xf*DSF2 or MeOH only. Download Figure S1, PDF file, 0.2 MB

Figure S2  GC-MS chromatograms for esterified natural extracts versus the 2-*cis*-hexadecenoic acid synthetic standard. (a) 2-*cis*-hexadecenoic acid; (b) RpfF extract; (c) WT extract; (d) RpfC extract. Download Figure S2, PDF file, 0.5 MB

Figure S3  Sequence identity (shown on black background) and similarity (shown on gray background) of *X. fastidiosa* (Xf) Tem1 RpfF and *P. agglomerans* (Pa) 299R F385_3254. The F385_3254 gene encodes a protein with 37% identity to that of *X. fastidiosa* RpfF. Download Figure S3, PDF file, 0.5 MB
