# Supplementary material for: Promiscuous Diffusible Signal Factor Production and Responsiveness of the Xylella fastidiosa Rpf System
Source: mBio. 2016 Jul 19;7(4):e01054-16. doi: 10.1128/mBio.01054-16 (PMC4958263; doi:10.1128/mBio.01054-16)
Supplement: Figure S1 — Induction of the XfDSF-biosensor strain by either 2 or 10 µl of a purified fraction of a DSF-containing extract of P. agglomerans 299R expressing X. fastidiosa RpfF (fractions named based on their retention times in minutes in HPLC [HPLC RT]). White bars represent induction of the sensor by 10 µM XfDSF2 or MeOH only. Download [file mbo004162902sf1.pdf]

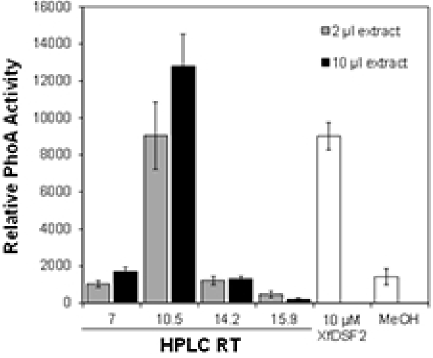

Figure S1. Induction of the *Xf*DSF biosensor by either 2 or 10 µl of a purified fraction of a DSF-containing extract of *P. agglomerans* 299R expressing *X. fastidiosa* RpfF (fractions named based on their retention times in minutes in HPLC [HPLC RT]). White bars represent induction of the sensor by 10 µM *Xf*DSF2 or MeOH only.
