# Supplementary material for: Promiscuous Diffusible Signal Factor Production and Responsiveness of the Xylella fastidiosa Rpf System
Source: mBio. 2016 Jul 19;7(4):e01054-16. doi: 10.1128/mBio.01054-16 (PMC4958263; doi:10.1128/mBio.01054-16)

Figure S2. GCMS chromatograms for esterified natural extracts versus the 2-cis-hexadecenoic acid synthetic standard. (a) 2-cis-hexadecenoic acid (b) RpfF extract (c) WT extract (d) RpfC extract

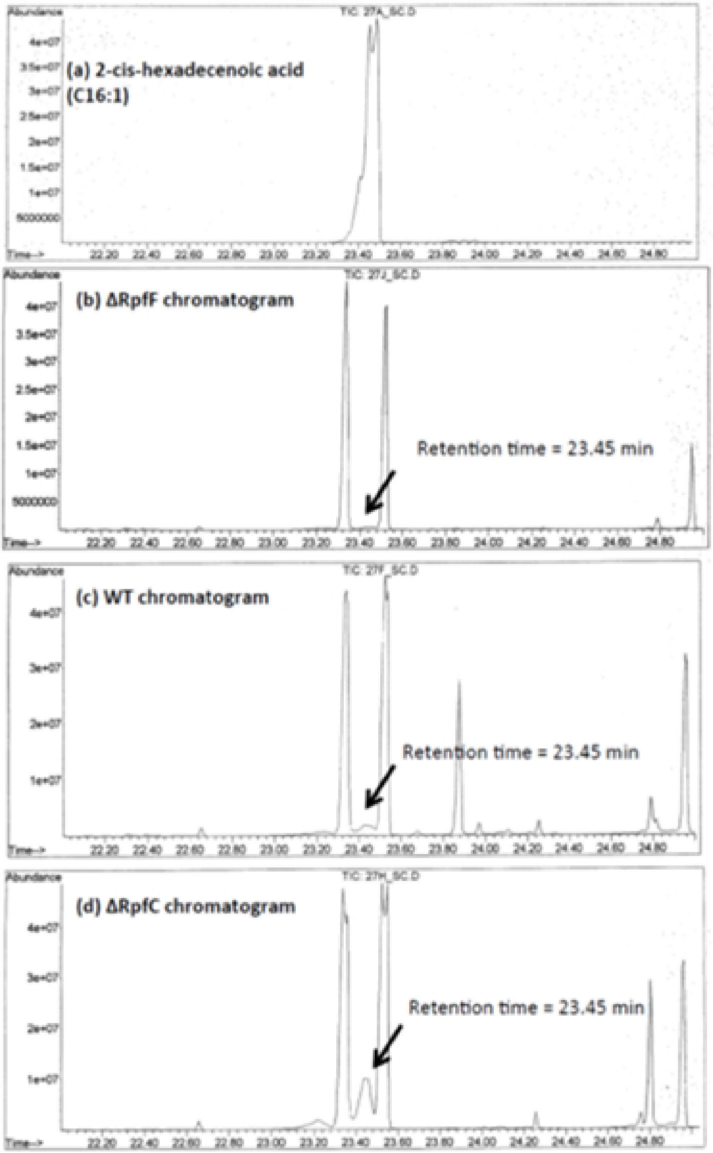

Supplement: Figure S2 — GC-MS chromatograms for esterified natural extracts versus the 2-cis-hexadecenoic acid synthetic standard. (a) 2-cis-hexadecenoic acid; (b) RpfF extract; (c) WT extract; (d) RpfC extract. Download [file mbo004162902sf2.pdf]
