# Supplementary material for: Promiscuous Diffusible Signal Factor Production and Responsiveness of the Xylella fastidiosa Rpf System
Source: mBio. 2016 Jul 19;7(4):e01054-16. doi: 10.1128/mBio.01054-16 (PMC4958263; doi:10.1128/mBio.01054-16)
Supplement: Figure S3 — Sequence identity (shown on black background) and similarity (shown on gray background) of X. fastidiosa (Xf) Tem1 RpfF and P. agglomerans (Pa) 299R F385_3254. The F385_3254 gene encodes a protein with 37% identity to that of X. fastidiosa RpfF. Download [file mbo004162902sf3.pdf]

```
Xf VLRQRLKEQTVQLPFVVLAS-DSNVFNLGGDLQLFCDLIRRKEREALLDYACRCVRGAYA
```

```
Xf MSAVHPIPHPICESSIRIIEETHRNVYWIYMHAHL---ARTTGAAYFSLKLIDDIMNYQS
Pa MIVIN---QATCRLFTEVGNTTQLVAYYEEGRRTYWMMLRAQPRPSFNHELIEEIMN---

Xf VLRQRLKEQTVQLPFVVLAS-DSNVFNLGGDLQLFCDLIRRKEREALLDYACRCVRGAYA
Pa -LSYAAQRSGLPIDFWVTGSLVPQMFNAGGDLRFFVECIRNNRREALRAYARACVDCIHS

Xf FHAGLNANVHSIALLQGNALGGGFEAALCCHTIVAEEGVMMGFPEVLFDLFPGMGAYSFM
Pa AARGFDTGAVTLAMIEGSALGGGFEAALAHHFILAQNNARMGFPEIAFNLFPGMGGYSLV

Xf RQRISPKLAERLILEGNLYSSEELLAIGLIDKVVPRGKGIEAVEQIIRDSKRRQYTWAAM
Pa ARRSGMKLAEELICEGESHSAEWYETRGLVDKVFQPGDSYRATRTFIDTLRPKLNGVRAM

Xf QEVKKIAHEVSLEEMIRITELWVDSALKLSNKSLRTMERLIRAQQTHKNTALK-N
Pa LKARQRVLQLSRAELMDITEDWVDYAFTIEPKDIAYMERLVQLQNRHSASLRKAG
```

Figure S3. Sequence identity (shade in black) and similarity (shade in grey) of *X. fastidiosa* Tem1 RpfF and *P. agglomerans* 299R F385_3254. F385_3254, encoding a protein with 37% identity to that of *X. fastidiosa* RpfF.
